# Post-Operative Chylothorax in Children Undergoing Congenital Heart Surgery

**DOI:** 10.7759/cureus.13811

**Published:** 2021-03-10

**Authors:** Mehnaz Atiq Ahmed

**Affiliations:** 1 Pediatric Cardiology, Department of Pediatrics, Liaquat National Hospital, Karachi, PAK

**Keywords:** chylothorax, post-operative complications, congenital heart surgery

## Abstract

Chylothorax is a rare postoperative complication of congenital heart surgery. It has high morbidity with increased hospital stay and cost of treatment. Damage to the thoracic duct, disruption of accessory lymphatic vessels, and increased venous pressure exceeding that in the thoracic duct have been proposed as the possible causes of chylothorax after surgery for congenital heart disease. Prompt diagnose with early initiation of treatment will reduce the duration of drainage. Staged treatment is the general principle in managing this serious complication. Loss of chyle leads to volume, nutritional and electrolyte depletion, immunological deficiencies and hematological complications. Identifying the underlying cause and addressing it is crucial to definitive management.

## Introduction and background

Chylothorax is a condition of chyle leakage from the lymphatic system into the pleural cavity [1]. Although there are numerous causes, chylothorax is a frequent and serious complication associated with congenital heart surgery, incidence being 0.5% to 6.5% [2,3]. A recent increase in the prevalence of post-operative chylothorax is due to increased performance of complex heart surgeries and possible early post-operative feeding [4]. It is associated with significant respiratory, nutritional, immunologic, hematologic, and metabolic morbidity and increased mortality [3,4].

## Review

Lymphatic system anatomy and physiology

The lymphatic system is composed of lymphatic vessels, lymph nodes, and associated lymphoid organs. It plays an integral role in the regulation of tissue fluid homeostasis, immune cell trafficking, and absorption of dietary fats.

Lymphatic vessels are blind-ended unidirectional absorptive vessels which transport interstitial fluid, immune cells, and macromolecules to the lymph nodes, and back to the blood circulation. The lymphatic vessels are found in almost every vascularized tissue except neural tissue and bone marrow [5].

**Figure 1:**
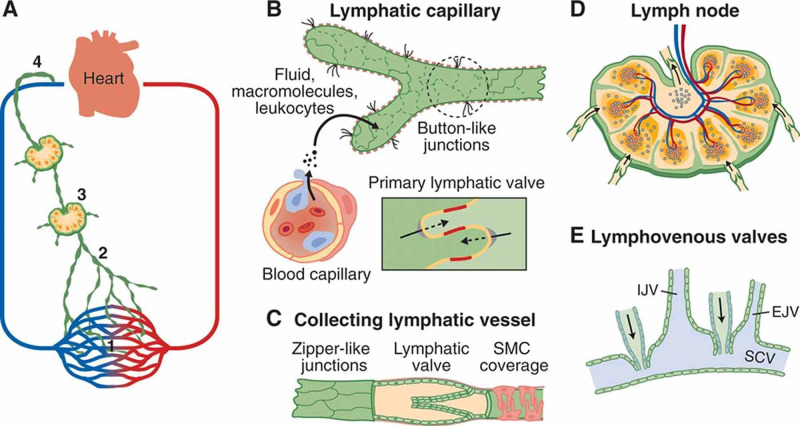
Anatomy of lymphatic vascular system. (A) Lymphatic vascular system consists of: 1. tissue lymphatic capillaries; 2. collecting vessels; 3. lymph nodes; 4. right thoracic duct. (B) Lymphatic capillaries absorb fluid through breaches endothelium and button-like junctions to form lymph. (C) collecting vessel with zipper-like junctions, valves and smooth muscle cells (SMC). (D) Lymph node with multiple afferent and one efferent vessel. (E) Lymph vessel provided with valves drain into internal jugular vein (IJV), external jugular vein (EJV) and subclavian vein (SCV). Adapted from Aspelund et al., Circulation Research. 2016; 118(3): 515–530 [5]. With permission.

Lymphatic capillaries converge into the larger collecting vessels which drain via chains of lymph nodes, opening eventually into the thoracic duct and the right lymphatic trunk which open into the venous circulation (Figure 1). There are eleven lymphatic trunks: gastrointestinal, lumbar, broncho-mediastinal, subclavian, jugular and descending intercostal. All, except for the gastrointestinal trunk, are paired [6]. Ultimate drainage of the lymphatic system is asymmetric. Right lymphatic duct drains lymph from right side of head, thorax, and right upper limb into right subclavian vein. The lymph from the rest of the body is drained by the thoracic duct. The thoracic duct originates from the cisterna chili at around L1 vertebral column and traverses up the aortic hiatus to join the left subclavian vein.

Understanding digestion and absorption of dietary fat is the key in formulating management plan of disorders of this process. Small- and medium-chain fatty acids are broken down into free fatty acids by intestinal enzymes. These are then absorbed into the portal circulation. Long-chain fatty acids are treated differently. They cannot be broken down and therefore fuse with lipids (phospholipids, cholesterol and cholesterol esters) to form chylomicrons. Chylomicrons are absorbed into the lymphatic channels of small intestine to form chyle.

Normal circulating volume of lymph is up to 2.5 liters per day [7]. It comprises of chyle and the interstitial fluid that is not drained by venous circulation. Lymph contains chylomicrons, lymphocytes (predominately T-lymphocytes), electrolytes, immunoglobulin, albumin, fibrinogen, glucose, fat-soluble vitamins, and antibodies. Causes of chylothorax in children are enlisted in Table 1 [8].

**Table 1:** Causes of chylothorax in children.

Congenital chylothorax	Traumatic	High venous pressures	Associated with tumors	Miscellaneous
(A) Congenital lymphatic malformation	(A) Surgical	Thrombosis of superior vena cava	Neurogenic tumors	(A) Granulomatous diseases
Lymphangiomatosis	Excision of lymph nodes	Deep vein thrombosis of upper extremity	Lymphoma	Tuberculosis
Lymphangiectasia	Congenital heart surgery	Post-operative Fontan Surgery	Teratoma	Histoplasmosis
Atresia of thoracic duct	Scoliosis operations		Wilm’s tumor	Sarcoidosis
(B) Associated with syndromes	Excision of vascular rings		Ovarian tumor	(B) Others
Down syndrome	Diaphragmatic hernia		Kaposi sarcoma	Staphyloccocal discitis
Turner’s syndrome	(B) Invasive diagnostic procedures			Henoch Schonlein purpura
Noonan’s syndrome	Subclavian vein catheterization			
Gorham-Stout syndrome	(C) Other trauma			
Yellow nail syndrome	Blunt or penetrating chest injury			
Hydrops fetalis	Thoracic spine surgery			

Etiology and risk factors for chylothorax after congenital heart surgery

Several mechanisms are responsible for post-operative complication of chylothorax: damage to the thoracic duct while cannulating superior vena cava during cardio-pulmonary bypass, surgical trauma to thoracic duct and/or disruption of accessory lymphatic vessels during dissection, increased central venous pressure (exceeding lymphatic pressure) after partial or complete cavo-pulmonary anastomosis and development of central venous thrombosis (both preventing drainage of the thoracic duct) [4,5]. Risk factors include [2,3] type of procedure and its complexity (1% for Risk Adjustment for Congenital Heart Surgery [RACHS] score 1 and 5.6% for RACHS score 5 and 6), low weight and height, duration of cardiopulmonary bypass and cross-clamping, and presence of syndromes, in particular Down syndrome, Noonan’s syndrome and Turner's Syndrome (the latter two are 4-7 times more likely to develop chylothorax due to lymphangiectasia, abnormal lymphatic collaterals and spontaneous chylothorax) [3,9-12]. Right ventricular dysfunction after total correction of tetralogy of Fallot predisposes to chylothorax by increasing right atrial central venous pressures [13]. Patients with Fontan procedure and heart transplant are at highest risk. An important cause of chylothorax is secondary chest closure which supports the etiology of non-specific mediastinal tissue damage and impaired post-operative hemodynamics [2]. Neonates are seven times more likely to develop chylothorax as compared to teenagers [3].

High volume centers have a low incidence according to one study. The difference may be due to better pre-operative patient selection, improved post-operative care and feeding protocols, differences in surgical techniques or may be differences in reporting chylothorax [3]. However, another study quotes the contrary [1].

Clinical features

The first sign of development of post-operative chylothorax is pleural fluid turning milky white in the chest tube. Sometimes chylothorax is serous, sanguineous or bloody [12]. It can develop from the 1st day up to 24th day after surgery [4] and can be unilateral or bilateral. However, in patients who are fasting post-operatively, effusions may appear serious. Pleural empyema can also produce opaque pleural fluid, as can pseudo-chylothorax (long-standing pleural effusion in which transudate becomes turbid due to accumulation of cholesterol and lecithin). The latter two can be distinguished by clinical features and laboratory investigations (see below) [8,14].

Without the chest tube, low volume chylothorax can be clinically silent. High volume collections can lead to dyspnea, cough, hypovolemic symptoms, and rarely, with rapid accumulation of fluid, may cause tension chylothorax. Since the accumulation is non-inflammatory, fever and pleuritic chest pain are not present [14].

Investigations

Investigations essentially focus on confirmation of chylothorax by fluid analysis and diagnosis of the cause. A persistent chest tube drainage of >5 ml/kg/day on 4th post-operative day or a milky nature of the fluid warrants investigation and management. Chest X-Ray or ultrasound may show unilateral or bilateral pleural effusion. Examination of fluid obtained by pleurocentesis will differentiate between chylothorax, pseudo-chylothorax and pleural empyema. Chyle will have high levels of triglycerides (>110 mg/dl or higher than serum triglycerides), proteins (>20 g/l), and a cholesterol content <200 mg/dl, absolute white cell count of >1,000/cumm with >80% of cells being lymphocytes [1,2,8,13,15]. A triglyceride content <50 mg/dl almost rules out chyle. Ambiguity exists when the level is between 50 mg/dl and 100 mg/dl. Lipoprotein electrophoresis which is considered to be a gold standard in diagnosing chylothorax should be considered in such a setting because rarely chylothorax may have low triglyceride levels [14]. Typical composition of chyle is given in Table 2 [8]. Pseudo-chylothorax, which is also milky, is characterized by a cholesterol concentration of >200 mg/dL, lower triglyceride composition (<110mg/dl), cholesterol/triglyceride ratio >1 and a pleural/serum cholesterol ratio >1 [8,16,17]. For prognostication certain laboratory investigations have been used by clinicians. These include serum C-reactive protein/pre-albumin ratio or their levels and transferrin as an acute phase reactant [16].

**Table 2:** Composition of chyle.

Components	Amount
pH	7.4-7.8
Absolute cell count	1,000 cells/L
Lymphocytes	400-6,800/cumm
Erythrocytes	50-600/cumm
Calories	200 Kcal/L
Total fat	0.4-0.8 g/dl
Cholesterol	65-200 mg/dl
Triglycerides	110 mg/dl (1.1 mmol/L)
Chylomicrons	present
Total protein	2-6 g/dl
Albumin	1.2-4.1 g/dl
Globulin	1.1-3.1 g/dl
Glucose	2.7-3.1 g/dl
Sodium	104-108 mmol/L
Potassium	3.4-5.0 mmol/L
Chloride	85-130 mmol/L
Calcium	3.4-6 mmol/L
Phosphate	0.8-4.2 mmol/L
Lactate dehydrogenase	<160 IU/L

Baseline echocardiography should be done to assess ventricular function, stenosis, or surgical site thrombosis. For unclear diagnosis, cardiac catheterization, computerized tomography scans, lymphangiography, lymphoscintigraphy or dynamic contrast-enhanced magnetic resonance lymphangiography may be helpful [8,10]. These specialized tests are done when conservative management fails and interventional treatment is planned.

Management of post-surgical chylothorax

Treatment of post-surgical chylothorax has two primary goals: relief of respiratory symptoms by drainage of fluid and prevent or reduce chyle collection in pleural space [12]. Management strategies for the second goal will depend upon the cause, volume and rate of accumulation of effusion, underlying disease and co-morbidities. The initial treatment in all cases is conservative and interventional therapy is reserved for refractory cases.

1. Conservative treatment

The goals of conservative management are to reduce chyle production by nutritional measures and relieve symptoms by image-guided chest tube drainage. This helps in re-expansion of lung, optimizes lung function and also guides treatment strategies. Sometimes drainage approximates lung and pleural surfaces, thereby sealing the leak.

Nutritional management should be aggressive with advice from a nutrition expert. Chylothorax diet aims at providing low long-chain triglycerides (because they undergo second esterification and enter lymphatic duct in the form of chylomicrons), and high medium-chain triglycerides (MCT, because they get coupled to albumin and directly enter portal circulation) either as oral or nasogastric tube feeding. A 10-day treatment with long-chain fatty acid-free MCT diet was found to be effective in 71% of patients in one study [2]. In case of oral intolerance best approach would be total parenteral nutrition (TPN). TPN is also recommended for high output of chyle or if central venous pressures are >15mmHg [2]. Fat-soluble vitamins, albumin or protein diet, electrolytes and calcium may be added as required.

Drugs may be added as indicated, like diuretics, sildenafil, angiotensin-converting enzyme (ACE) inhibitors, or heparin for thrombosis. Cardiac catheterization may be needed to document increased venous pressures and address stenosis with balloon dilatation or placement of stents [2].

**Figure 2:**
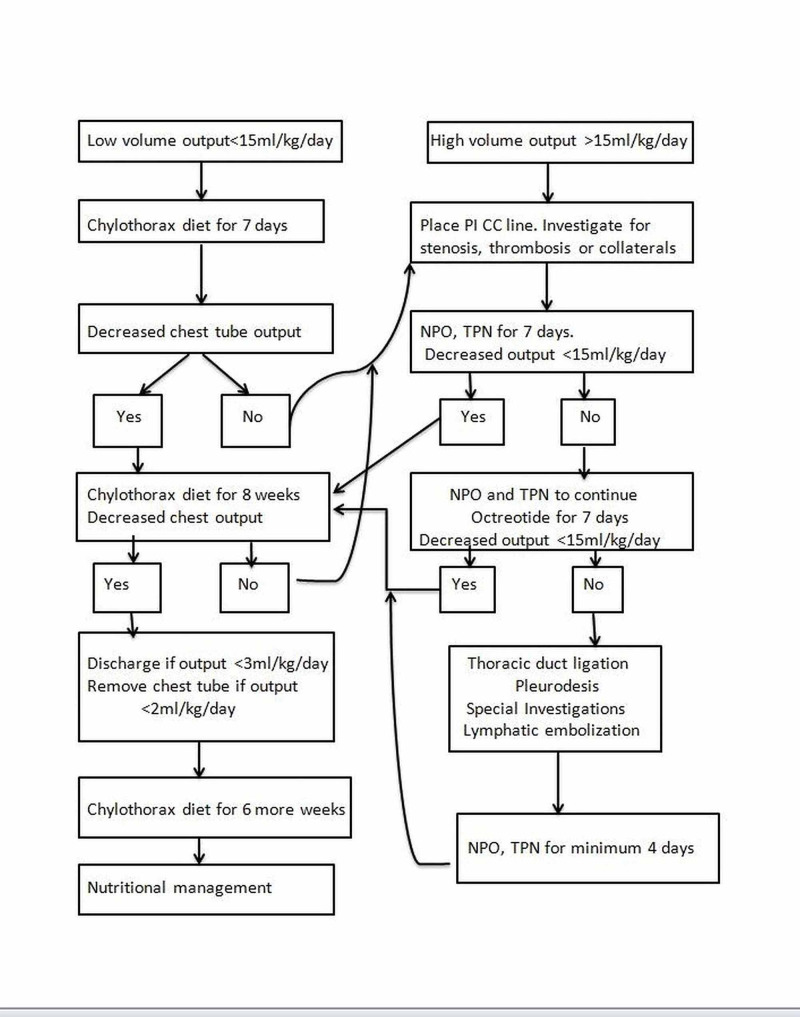
Management guidelines for post-operative chylothorax. Abbreviations: PICC: peripherally inserted central catheter; NPO: nil per oral; TPN: total parenteral nutrition.

Figure 2 is a guiding algorithm to an overall management approach. Management is in phases and escalation of treatment is decided by the chyle output and number of days of treatment. First phase is nutritional management for five to seven days. If chyle production exceeds 15 ml/kg/day (or 20 ml/kg/day) [18], the 2nd phase of treatment would be to stop oral feeds and provide TPN for five to seven days. If TPN fails to reduce chylous output then the 3rd phase of a five to seven days trial of drugs is initiated. Previously steroids were used but recent protocols have not included it. All protocols use intravenous infusion or subcutaneous boluses of octreotide, with a starting dose of 0.5-4 mcg/kg/hr or 10 mcg/kg/day in three divided doses, increasing 5-10 mcg/kg/day every 72-96 hours, maximum of 40 mcg/kg/day. Indication for starting octreotide is a chylous drainage for >2 weeks or drain output of >15 ml/kg/day [4]. Octreotide, a somatostatin analogue, reduces lymph formation by directly acting on vascular somatostatin receptors and indirectly by reducing intestinal blood flow and motility. Adverse reactions are mild and include abdominal distension, hypoalbuminemia and rarely may contribute to septicemia through its inhibitory role on immune responses [4]. Duration of treatment with octreotide is generally for five to seven days and then weaning off over four days [4,2,12,19].

Success of any conservative regimen is when the drainage output becomes <2 ml/kg/day. Throughout the treatment, chylothorax nutritional management should be continued. Even after reduction in chyle output, dietary management with MCT diet should be continued for 6-8 weeks and with low-fat diet for another six weeks [15].

Majority of patients, up to 80%-85%, respond to conservative treatment [9,20]. Treatment failure with octreotide warrants further investigations and interventional treatment with weaning off of the drug at 25% dose daily in four days [2].

2. Interventional treatment

Patients who fail to respond to conservative treatment have the option of surgical or interventional treatment. Surgical treatment reduces mortality from 50% to 10%. Indication for surgical treatment include chyle production exceeding 100 ml/kg/day or 100 ml/year of age for five days, persistent chyle drainage of >100 ml/day for >2 weeks despite conservative management, unchanged drainage output for one to two weeks [14,15], or clinical deterioration (hemodynamic, nutritional, immunological or metabolic).

Early reoperation for chylothorax may put anastomosis at risk and conservative treatment for two to four2-4 weeks is therefore recommended. However early surgical treatment is recommended in small children with high volume losses due to their delicate fluid and electrolyte balance.

a: Surgical Treatment

Direct surgical ligation of thoracic duct is done from above the diaphragm between T8 and T12. After ligation, the lymph drains via lymphatic collaterals and lympho-venous anastomoses. The challenge is identification of the thoracic duct or the leakage site which can be made prominent by giving cream intra-operatively by nasogastric tube. Thoracoscopic ligation of the thoracic duct has also been done. If leakage site is not identified, then mass ligation of the thoracic duct and tissue around it, aorta, azygous vein and esophagus is done or ligation of cisterna chyli may also be helpful [8,14]. Thoracic duct ligation is successful in 90% of cases [15].

Pleurodesis, a procedure involving chemical obliteration of pleural space using talc, tetracyclin, bleomycin or povidone-iodine, may be successful in patients who continue to produce chyle in large amounts after surgery [8]. Pleuro-peritoneal shunt or external intermittent drainage are other options for refractory patients whose thoracic duct ligation has failed.

b: Interventional Radiological Treatment

Expertise in this field is very limited and therefore it is recommended in refractory cases of chylothorax. Lymphangiography (conventional or magnetic resonance) outlines the thoracic duct and the leakage site. Embolization is done through micro-catheters using ethiodized oil (lipiodol), endovascular coils and n-butyl cyanoacrylate glue, alone or in combination [10,21]. After successful thoracic duct embolization, short-term complications noted are hypotension, systemic inflammatory response syndrome, pulmonary edema and rarely procedure-related stroke [2]. Delayed complications may be seen like chronic diarrhea and lymphedema of lower extremities [19,22,23].

Morbidity from post-surgical chylothorax 

The impact of chylothorax is considerable because it increases morbidity and puts patients twice at risk of dying as compared to patients who do not develop chylothorax [6]. Delayed diagnosis correlates with longer duration of chest tube drainage [13]. Chyle leak, proportional to its volume, leads to volume depletion, lymphopenia, hypoalbuminemia, loss of lipids, electrolytes which would lead to a catabolic state and malnutrition, immunologic deficiencies, metabolic and hematological complications, all having a detrimental effect on an already compromised post-operative state [3,4]. Lymphopenia is an absolute peripheral lymphocyte count of <1500/dl and directly correlates with duration of chylothorax [15].

There is a reported increased risk of sepsis due to the bacteriostatic properties of lecithin and fatty acids in the chyle as well as decrease in cellular and humoral immunity (hypogammaglobulinemia). There is an increased loss of anti-thrombin and fibrinogen, the former causing increased risk of thrombosis and the latter bleeding diathesis [15]. Electrolyte loss leads to hyponatremia, hypocalcemia and metabolic acidosis.

Large effusions compromise lung function, which is relevant in patients with single ventricle physiology. In patients with Fontan surgery, plastic bronchitis is a frequent comorbidity associated with chylothorax, both related to abnormal pulmonary lymphatic perfusion [10]. Long-term complications of chylothorax in neonates and children have not been reported [20,21].

## Conclusions

Chylothorax is a rare complication of congenital heart surgery. It significantly increases morbidity and mortality, cost of treatment and length of hospital stay. Early diagnosis and prompt initiation of treatment may lead to early resolution. There is no overall consensus on best management protocol or therapeutic strategies. Different algorithms reported reflect Institutions or physicians experience and preference. Mainstay of treatment is conservative with MCT diet and dietary supplements. Other treatment modalities if needed also have a high success rate. Prevention may be important by careful patient selection in single ventricular physiology, creating fenestration in Fontan circulation and foramen ovale in anticipated post-operative right atrial hypertension and aggressive medical management of restrictive right ventricular physiology in tetralogy of Fallot.
